# Supplementary material for: Personal approach for cancer treatment: A meta-analysis of Phase II clinical trials
Source: PLoS One. 2025 Sep 26;20(9):e0332599. doi: 10.1371/journal.pone.0332599 (PMC12469113; doi:10.1371/journal.pone.0332599)
Supplement: S2 Table — (PDF) [file pone.0332599.s002.pdf]

S3 Table. Assessment of Study Quality

**Risk of bias**	**Arms (n=81)**	**Trials (n=50)**
High	6 (7.4%)	6 (12%)
Moderate	20 (24.7%)	20 (40%)
Low	55 (67.9%)	24 (48%)
