# Supplementary material for: Personal approach for cancer treatment: A meta-analysis of Phase II clinical trials
Source: PLoS One. 2025 Sep 26;20(9):e0332599. doi: 10.1371/journal.pone.0332599 (PMC12469113; doi:10.1371/journal.pone.0332599)
Supplement: S3 Table — Grade approach. (PDF) [file pone.0332599.s003.pdf]

S4 Table. Assessment of Study Quality. GRADE Approach

Parameter	Number of participans	Number of studies	GRADE (Certainty of Evidence)
**RR: Response Rate [%]**	2767	26	High (++++)
**PFS: median survival, [months]**	4367	35	High (++++)
**1-year PFS rates [%]**			
**OS: median survival, [months]**	4903	42	High (++++)
**1-year OS rates [%]**			
