# Supplementary material for: Personal approach for cancer treatment: A meta-analysis of Phase II clinical trials
Source: PLoS One. 2025 Sep 26;20(9):e0332599. doi: 10.1371/journal.pone.0332599 (PMC12469113; doi:10.1371/journal.pone.0332599)
Supplement: S6 File — https://github.com/MikhailPot/PreciseOnco_meta-analysis. (PDF) [file pone.0332599.s006.pdf]

````
```{r setup, include=FALSE}

#load libraries

library(tidyverse)

library(meta)

library(metafor)

#upload data


tb <- read.csv2("E:/Статьи и НИР/Meta-analysis/Обработка данных/Dataset_git_repo.csv", dec = ",")

# meta-analysis variable DerSimonanian-Laird algotrithm

RR_out <- metaprop(RR, N, sm="PFT", data=tb, method="Inverse", method.tau="DL") #Response Rate (RR)

RR_out


tb_OS = tb[!is.na(tb$OS2),]

OS_out <- metaprop(OS2, N, sm="PFT", data=tb, method="Inverse", method.tau="DL") #Overall survival (OS)

OS_out

PFS_out <- metaprop(PFS2, N, sm="PFT", data=tb, method="Inverse", method.tau="DL") #Progression-free survival (PFS)

PFS_out

```{r pressure, echo=FALSE}

#import libraries for forestplot

library(forestploter)

library(grid)

```{r pressure, echo=FALSE}

#OS meta-regression & forestplot

parameters <- c("Genomic markers", "Randomisation", "Single/multi-central", "Pre-treated", "Target/Immunotherapy", "Chemotherapy", "Number of patients per arm > 40",  "Journal 5 year IF > 10")

Est_OS = c(metareg(OS_out, ~ G1_1)$beta[2], metareg(OS_out, ~ Rand)$beta[2], metareg(OS_out, ~ Cent)$beta[2], metareg(OS_out, ~ Ptrt)$beta[2], metareg(OS_out, ~ target)$beta[2], metareg(OS_out, ~ chemo.t)$beta[2], metareg(OS_out, ~ N_count)$beta[2],  metareg(OS_out, ~ IF)$beta[2])


Se_OS = c(metareg(OS_out, ~ G1_1)$se[2], metareg(OS_out, ~ Rand)$se[2], metareg(OS_out, ~ Cent)$se[2], metareg(OS_out, ~ Ptrt)$se[2], metareg(OS_out, ~ target)$se[2], metareg(OS_out, ~ chemo.t)$se[2], metareg(OS_out, ~ N_count)$se[2],  metareg(OS_out, ~ IF)$se[2])
````
````

````
Ci_lb_OS = c(metareg(OS_out, ~ G1_1)$ci.lb[2], metareg(OS_out, ~ Rand)$ci.lb[2], metareg(OS_out, ~ Cent)$ci.lb[2], metareg(OS_out, ~ Ptrt)$ci.lb[2], metareg(OS_out, ~ target)$ci.lb[2], metareg(OS_out, ~ chemo.t)$ci.lb[2], metareg(OS_out, ~ N_count)$ci.lb[2],  metareg(OS_out, ~ IF)$ci.lb[2])


Ci_ub_OS = c(metareg(OS_out, ~ G1_1)$ci.ub[2], metareg(OS_out, ~ Rand)$ci.ub[2], metareg(OS_out, ~ Cent)$ci.ub[2], metareg(OS_out, ~ Ptrt)$ci.ub[2], metareg(OS_out, ~ target)$ci.ub[2], metareg(OS_out, ~ chemo.t)$ci.ub[2], metareg(OS_out, ~ N_count)$ci.ub[2],  metareg(OS_out, ~ IF)$ci.ub[2])


Pval_OS = c(metareg(OS_out, ~ G1_1)$pval[2], metareg(OS_out, ~ Rand)$pval[2], metareg(OS_out, ~ Cent)$pval[2], metareg(OS_out, ~ Ptrt)$pval[2], metareg(OS_out, ~ target)$pval[2], metareg(OS_out, ~ chemo.t)$pval[2], metareg(OS_out, ~ N_count)$pval[2],  metareg(OS_out, ~ IF)$pval[2])


fr_frame_OS <- data.frame(parameters)


fr_frame_OS$` ` <- paste(rep("  ", 8), collapse = "  ")

fr_frame_OS[3:7] <- c(Pval_OS, Est_OS, Se_OS, Ci_lb_OS, Ci_ub_OS)


fr_frame_OS[3:7] <- round(fr_frame_OS[3:7], 3)

#fr_frame_OS[1] <- round(fr_frame_OS[1], 3)

#fr_frame_OS[1] <- parameters

colnames(fr_frame_OS) <- c("Parameters", "  ", "P-value")


forest(fr_frame_OS[,1:3], fr_frame_OS[,4], fr_frame_OS[,6], fr_frame_OS[,7], fr_frame_OS[,5], ci_column = 2, xlim = c(-0.5, 0.5), ticks_at = c(-0.5, 0,  0.5), theme = tm, ci_pch = 25, ref_line = 0)


#write.csv2(fr_frame_OS, "E:/Статьи и НИР/Meta-analysis/Обработка данных/Tables_out/metareg_forest_OS_r.csv")

```{r pressure, echo=FALSE}

#PFS meta-regression & forestplot


parameters <- c("Genomic markers", "Randomisation", "Single/multi-central", "Pre-treated", "Target/Immunotherapy", "Chemotherapy", "Number of patients per arm > 40",  "Journal 5 year IF > 10")

Est_PFS = c(metareg(PFS_out, ~ G1_1)$beta[2], metareg(PFS_out, ~ Rand)$beta[2], metareg(PFS_out, ~ Cent)$beta[2], metareg(PFS_out, ~ Ptrt)$beta[2], metareg(PFS_out, ~ target)$beta[2], metareg(PFS_out, ~ chemo.t)$beta[2], metareg(PFS_out, ~ N_count)$beta[2],  metareg(PFS_out, ~ IF)$beta[2])
````
````

````
Se_PFS = c(metareg(PFS_out, ~ G1_1)$se[2], metareg(PFS_out, ~ Rand)$se[2], metareg(PFS_out, ~ Cent)$se[2],
metareg(PFS_out, ~ Ptrt)$se[2], metareg(PFS_out, ~ target)$se[2], metareg(PFS_out, ~ chemo.t)$se[2],
metareg(PFS_out, ~ N_count)$se[2],  metareg(PFS_out, ~ IF)$se[2])


Ci_lb_PFS = c(metareg(PFS_out, ~ G1_1)$ci.lb[2], metareg(PFS_out, ~ Rand)$ci.lb[2], metareg(PFS_out, ~
Cent)$ci.lb[2], metareg(PFS_out, ~ Ptrt)$ci.lb[2], metareg(PFS_out, ~ target)$ci.lb[2], metareg(PFS_out, ~
chemo.t)$ci.lb[2], metareg(PFS_out, ~ N_count)$ci.lb[2],  metareg(PFS_out, ~ IF)$ci.lb[2])


Ci_ub_PFS = c(metareg(PFS_out, ~ G1_1)$ci.ub[2], metareg(PFS_out, ~ Rand)$ci.ub[2], metareg(PFS_out, ~
Cent)$ci.ub[2], metareg(PFS_out, ~ Ptrt)$ci.ub[2], metareg(PFS_out, ~ target)$ci.ub[2], metareg(PFS_out, ~
chemo.t)$ci.ub[2], metareg(PFS_out, ~ N_count)$ci.ub[2],  metareg(PFS_out, ~ IF)$ci.ub[2])


Pval_PFS = c(metareg(PFS_out, ~ G1_1)$pval[2], metareg(PFS_out, ~ Rand)$pval[2], metareg(PFS_out, ~
Cent)$pval[2], metareg(PFS_out, ~ Ptrt)$pval[2], metareg(PFS_out, ~ target)$pval[2], metareg(PFS_out, ~
chemo.t)$pval[2], metareg(PFS_out, ~ N_count)$pval[2],  metareg(PFS_out, ~ IF)$pval[2])


fr_frame_PFS <- data.frame(parameters)


fr_frame_PFS$` ` <- paste(rep("  ", 8), collapse = "  ")

fr_frame_PFS[3:7] <- c(Pval_PFS, Est_PFS, Se_PFS, Ci_lb_PFS, Ci_ub_PFS)


fr_frame_PFS[3:7] <- round(fr_frame_PFS[3:7], 3)

#fr_frame_PFS[1] <- round(fr_frame_PFS[1], 3)

#fr_frame_PFS[1] <- parameters

colnames(fr_frame_PFS) <- c("Parameters", "  ", "P-value")


forest(fr_frame_PFS[,1:3], fr_frame_PFS[,4], fr_frame_PFS[,6], fr_frame_PFS[,7], fr_frame_PFS[,5], ci_column =
2, xlim = c(-0.5, 0.5), ticks_at = c(-0.5, 0,  0.5), theme = tm, ci_pch = 25, ref_line = 0)


#write.csv2(fr_frame_PFS, "E:/Статьи и НИР/Meta-analysis/Обработка
данных/Tables_out/metareg_forest_PFS_r.csv")

```{r pressure, echo=FALSE}

#RR meta-regression & forestplot

parameters <- c("Genomic markers", "Randomisation", "Single/multi-central", "Pre-treated",
"Target/Immunotherapy", "Chemotherapy", "Number of patients per arm > 40",  "Journal 5 year IF > 10")
````
````

```
Est_RR = c(metareg(RR_out, ~ G1_1)$beta[2], metareg(RR_out, ~ Rand)$beta[2], metareg(RR_out, ~ Cent)$beta[2], metareg(RR_out, ~ Ptrt)$beta[2], metareg(RR_out, ~ target)$beta[2], metareg(RR_out, ~ chemo.t)$beta[2], metareg(RR_out, ~ N_count)$beta[2],  metareg(RR_out, ~ IF)$beta[2])

Se_RR = c(metareg(RR_out, ~ G1_1)$se[2], metareg(RR_out, ~ Rand)$se[2], metareg(RR_out, ~ Cent)$se[2], metareg(RR_out, ~ Ptrt)$se[2], metareg(RR_out, ~ target)$se[2], metareg(RR_out, ~ chemo.t)$se[2], metareg(RR_out, ~ N_count)$se[2],  metareg(RR_out, ~ IF)$se[2])

Ci_lb_RR = c(metareg(RR_out, ~ G1_1)$ci.lb[2], metareg(RR_out, ~ Rand)$ci.lb[2], metareg(RR_out, ~ Cent)$ci.lb[2], metareg(RR_out, ~ Ptrt)$ci.lb[2], metareg(RR_out, ~ target)$ci.lb[2], metareg(RR_out, ~ chemo.t)$ci.lb[2], metareg(RR_out, ~ N_count)$ci.lb[2],  metareg(RR_out, ~ IF)$ci.lb[2])

Ci_ub_RR = c(metareg(RR_out, ~ G1_1)$ci.ub[2], metareg(RR_out, ~ Rand)$ci.ub[2], metareg(RR_out, ~ Cent)$ci.ub[2], metareg(RR_out, ~ Ptrt)$ci.ub[2], metareg(RR_out, ~ target)$ci.ub[2], metareg(RR_out, ~ chemo.t)$ci.ub[2], metareg(RR_out, ~ N_count)$ci.ub[2],  metareg(RR_out, ~ IF)$ci.ub[2])

Pval_RR = c(metareg(RR_out, ~ G1_1)$pval[2], metareg(RR_out, ~ Rand)$pval[2], metareg(RR_out, ~ Cent)$pval[2], metareg(RR_out, ~ Ptrt)$pval[2], metareg(RR_out, ~ target)$pval[2], metareg(RR_out, ~ chemo.t)$pval[2], metareg(RR_out, ~ N_count)$pval[2],  metareg(RR_out, ~ IF)$pval[2])

fr_frame_RR <- data.frame(parameters)

fr_frame_RR$` ` <- paste(rep("  ", 8), collapse = "  ")
fr_frame_RR[3:7] <- c(Pval_RR, Est_RR, Se_RR, Ci_lb_RR, Ci_ub_RR)

fr_frame_RR[3:7] <- round(fr_frame_RR[3:7], 3)
#fr_frame_RR[1] <- round(fr_frame_RR[1], 3)
#fr_frame_RR[1] <- parameters
colnames(fr_frame_RR) <- c("Parameters", "  ", "P-value")

forest(fr_frame_RR[,1:3], fr_frame_RR[,4], fr_frame_RR[,6], fr_frame_RR[,7], fr_frame_RR[,5], ci_column = 2, xlim = c(-0.5, 0.5), ticks_at = c(-0.5, 0,  0.5), theme = tm, ci_pch = 25, ref_line = 0)

#write.csv2(fr_frame_RR, "E:/Статьи и НИР/Meta-analysis/Обработка данных/Tables_out/metareg_forest_RR_r.csv")
```

```{r pressure, echo=FALSE}
```

#Mann-Whitney test code example. The analysis was performed with wilcox.test command with parameters paired = F and correct = F. The example provided for PFS. The similar analysis was performed for OS and RR.

```
wilcox.test(tb2G[,1]~tb2G[,11])

wilcox.test(tb_OS["OS"]~tb_OS["G1_1"])

wilcox.test(as.numeric(unlist(tb_OS["OS"]))~as.numeric(unlist(tb_OS["G1_1"])), paired = F, correct = F)


wilcox.test(as.numeric(unlist(tbb["PFS_2.."]))~as.numeric(unlist(tbb["G1_1"])), paired = F, correct = F)

wilcox.test(as.numeric(unlist(tbb["PFS_2.."]))~as.numeric(unlist(tbb["Rand"])), paired = F, correct = F)

wilcox.test(as.numeric(unlist(tbb["PFS_2.."]))~as.numeric(unlist(tbb["Cent"])), paired = F, correct = F)

wilcox.test(as.numeric(unlist(tbb["PFS_2.."]))~as.numeric(unlist(tbb["Ptrt"])), paired = F, correct = F)

wilcox.test(as.numeric(unlist(tbb["PFS_2.."]))~as.numeric(unlist(tbb["target"])), paired = F, correct = F)

wilcox.test(as.numeric(unlist(tbb["PFS_2.."]))~as.numeric(unlist(tbb["N_count"])), paired = F, correct = F)

wilcox.test(as.numeric(unlist(tbb["PFS_2.."]))~as.numeric(unlist(tbb["IF"])), paired = F, correct = F)
```

#In case of normal distribution we performed Student test, with parameters paired = F and correct = F.

#Distribution was tested with Shapiro-Wilk test

````
```{r pressure, echo=FALSE}

ffm = read.csv2("E:/Статьи и НИР/Meta-analysis/Обработка данных/true_med.csv", dec = ",")

shapiro.test(ffm[ffm[,4]== "0", ][,3])

wilcox.test(ffm[,1]~ffm[,4], paired = F, correct = F)
```
````
