# Supplementary material for: Personal approach for cancer treatment: A meta-analysis of Phase II clinical trials
Source: PLoS One. 2025 Sep 26;20(9):e0332599. doi: 10.1371/journal.pone.0332599 (PMC12469113; doi:10.1371/journal.pone.0332599)
Supplement: Attachment — Submitted filename: Response to reviewers.docx [file pone.0332599.s008.docx]

We thank the reviewers and the editor for the crucial comments that will improve our manuscript.

The supplements were revised and corrected following the journal’s data availability policy. We added additional information on the Risk of bias evaluation and provided the reasons for exclusion for all the selected trials. We also added supportive information on the data extraction process (names of the reviewers and date of extraction). The dataset and the information on excluded and included studies were revised and corrected following the journal's requirements (S4 Dataset, S5 File [https://www.doi.org/10.5281/zenodo.10966214](https://www.doi.org/10.5281/zenodo.10966214%20)). The results of the certainty of evidence assessment (GRADE approach) are provided in S3 Table, and the method description is included on p.7.

Please find below our detailed responses to each point raised by reviewers.

***Responses to Reviewer 1.***

Reviewer 1 commented: “Clarify the methodology regarding the selection criteria for included studies, particularly how studies with mixed treatment types were handled”.

Response: We added additional information in the Methodology section to clarify the selection criteria, especially for mixed studies: The corrections were made in the 1^st^ paragraphs of the “Eligibility Criteria and Study Selection Strategy” section on p.5 (“Studies of chemotherapy, immunotherapy, targeted therapy, and multi-agent trials, including various treatments, were selected.”) and the “Data Synthesis and Statistics” section on p.7 (“We considered multi-agent therapy as targeted or immune-oncological treatment if those were appointed in combination with other treatments.”).

Reviewer 1 suggested: “Expand on the limitations of the study, especially concerning the heterogeneity of included trials and the potential variability in genetic markers across studies”.

Response: The study limitations section was extended with a focus on study heterogeneity on page 15 (Only phase II clinical trial data collected from the PubMed database is a limitation of the study. The studies varied in genomic markers and demonstrated a high level of heterogeneity across RR, OS, and PFS. Oncological diseases are quite heterogeneous in general; RR, OS, and PFS depend on cancer type, disease stage, and treatment. Future analysis may include Phase III clinical trials, collected from various databases with an extended subgroup analysis and an assessment of cost-effectiveness and quality-of-life outcomes.”).

Reviewer 1: “Consider a brief discussion on the implications of journal impact factor, even if found non-significant, as this adds context to the study design evaluation”.

Response: We included a discussion on the implications of the journal impact factor on page 14 (IF was evaluated as an additional parameter to assess study variability and quality. The non-significant results of the meta-regression and subgroup analysis suggest comparable RR, OS, and PFS outcomes across studies published in both high-impact and other journals. These findings indicate that the ability of genomics-based personalization to improve treatment outcomes is consistently reported, regardless of journal impact.”.

Reviewer 1 commented: “Include additional details on the statistical tests used (e.g., assumptions for the Mann-Whitney test) to ensure reproducibility”.

Response: We added additional information on the statistical tests and clarified the conditions for their use on page 7 (“We compared the effects using the Chi-square test for categorical variables and employed the t-test or Mann-Whitney test based on the type of distribution (normal or non-normal) for continuous variables (RR, OS, and PFS), with a significance level set at p<0.05.”).

***Responses to Reviewer 2***

Reviewer 2 commented: “First, the study reports considerable heterogeneity across key outcomes (RR, PFS, OS). Conducting subgroup analyses (e.g., by cancer type, treatment modality, or geographical region) and sensitivity analyses could help identify and address potential sources of heterogeneity.”

Response: We agreed that high heterogeneity is a limitation of the study and included several sentences to highlight it in the 2^nd^ paragraph of page 15 (“The studies varied in genomic markers and demonstrated a high level of heterogeneity across RR, OS, and PFS.”). Thank you for the suggestion that potential reasons for such results may be associated with various cancer types and treatments. Oncological diseases are quite heterogeneous in general; RR, OS, and PFS depend on cancer type, disease stage, and treatment. We described this topic in the discussion section, extending the limitations on page 15 (“Oncological diseases are quite heterogeneous in general; RR, OS, and PFS depend on cancer type, disease stage, and treatment.”). We also enhanced the explanation of heterogeneity on page 10 (“High heterogeneity rates may be associated with different response absolute metrics of outcomes for various cancers, disease stages, and treatments.”).

Reviewer 2 commented: “Second, the data collection is restricted to PubMed, which may lead to the exclusion of relevant studies. Expanding the search to include additional databases, such as EMBASE or Cochrane Library, would ensure a more comprehensive literature review.”

Response: We underlined such study limitations as PubMed and Phase II clinical trial restricted data search on page 15 (“Only Phase II clinical trial data collected from the PubMed database is a limitation of the study.”).

Reviewer 2 commented: “Lastly, as the analysis focuses exclusively on Phase II trials, the clinical applicability of the findings may be limited. Including Phase III trial data or real-world evidence, along with an assessment of cost-effectiveness and quality-of-life outcomes, could significantly enhance the practical relevance and impact of the study's conclusions”.

Response: We agree that the recommendations provided will make the study more informative and impactful, however, it will require reorganization of the manuscript after full re-analysis in accordance with the PRISMA workflow. This manuscript is the first step in genomics-based personalization analysis, and our current research includes the extended analysis suggested by the reviewer. A consideration of future research was included in the revised version of the manuscript on page 15 (“Future analysis may include Phase III clinical trials, collected from various databases with an extended subgroup analysis and an assessment of cost-effectiveness and quality-of-life outcomes.”).
